# Supplementary material for: Iterative improvement in the automatic modular design of robot swarms
Source: PeerJ Comput Sci. 2020 Dec 7;6:e322. doi: 10.7717/peerj-cs.322 (PMC7924708; doi:10.7717/peerj-cs.322)
Supplement: Supplemental Information 3 [file peerj-cs-06-322-s003.zip › argos3/doc/api/standalone/a00428_source.html]

ARGoS: plugins/robots/eye-bot/simulator/eyebot\_proximity\_default\_sensor.cpp Source File


- Main Page
- Related Pages
- Namespaces
- Classes
- Files

- File List
- File Members

# plugins/robots/eye-bot/simulator/eyebot\_proximity\_default\_sensor.cpp

Go to the documentation of this file.

```
00001 
00007 #include <argos3/core/simulator/entity/embodied_entity.h>
00008 #include <argos3/core/simulator/entity/composable_entity.h>
00009 #include <argos3/core/simulator/simulator.h>
00010 
00011 #include "eyebot_proximity_default_sensor.h"
00012 
00013 namespace argos {
00014 
00015    /****************************************/
00016    /****************************************/
00017 
00018    class CProximitySensorImpl : public CProximityDefaultSensor {
00019 
00020    public:
00021 
00022       virtual Real CalculateReading(Real f_distance) {
00023          if(f_distance < 0.009889556) {
00024             return 1.0;
00025          }
00026          else {
00027             return 0.0100527 / (f_distance + 0.000163144);
00028          }
00029       }
00030 
00031    };
00032 
00033    /****************************************/
00034    /****************************************/
00035 
00036    CEyeBotProximityDefaultSensor::CEyeBotProximityDefaultSensor() :
00037       m_pcProximityImpl(new CProximitySensorImpl()) {}
00038 
00039    /****************************************/
00040    /****************************************/
00041 
00042    CEyeBotProximityDefaultSensor::~CEyeBotProximityDefaultSensor() {
00043       delete m_pcProximityImpl;
00044    }
00045 
00046    /****************************************/
00047    /****************************************/
00048 
00049    void CEyeBotProximityDefaultSensor::SetRobot(CComposableEntity& c_entity) {
00050       try {
00051          m_pcProximityImpl->SetRobot(c_entity);
00052       }
00053       catch(CARGoSException& ex) {
00054          THROW_ARGOSEXCEPTION_NESTED("Can't set robot for the eye-bot proximity default sensor", ex);
00055       }
00056    }
00057 
00058    /****************************************/
00059    /****************************************/
00060 
00061    void CEyeBotProximityDefaultSensor::Init(TConfigurationNode& t_tree) {
00062       m_pcProximityImpl->Init(t_tree);
00063    }
00064 
00065    /****************************************/
00066    /****************************************/
00067 
00068    void CEyeBotProximityDefaultSensor::Update() {
00069       m_pcProximityImpl->Update();
00070       for(size_t i = 0; i < 24; ++i) {
00071          m_tReadings[i].Value = m_pcProximityImpl->GetReadings()[i];
00072       }
00073    }
00074 
00075    /****************************************/
00076    /****************************************/
00077 
00078    void CEyeBotProximityDefaultSensor::Reset() {
00079       m_pcProximityImpl->Reset();
00080    }
00081 
00082    /****************************************/
00083    /****************************************/
00084 
00085    REGISTER_SENSOR(CEyeBotProximityDefaultSensor,
00086                    "eyebot_proximity", "default",
00087                    "Carlo Pinciroli [ilpincy@gmail.com]",
00088                    "1.0",
00089                    "The eye-bot proximity sensor.",
00090                    "This sensor accesses the eye-bot proximity sensor. For a complete description\n"
00091                    "of its usage, refer to the ci_eyebot_proximity_sensor.h interface. For the XML\n"
00092                    "configuration, refer to the default proximity sensor.\n",
00093                    "Usable"
00094                   );
00095 
00096 }
```

---

Generated on 10 Jul 2018 for ARGoS by 
 1.6.1
